# Higher Rates of Low Socioeconomic Status, Marginalization, and Stress in Black Transgender Women Compared to Black Cisgender MSM in The MARI Study

**DOI:** 10.3390/ijerph18042183

**Published:** 2021-02-23

**Authors:** Jonathan S. Russell, DeMarc A. Hickson, Liadh Timmins, Dustin T. Duncan

**Affiliations:** 1 Department of Epidemiology and ICAP, Columbia University Mailman School of Public Health, New York, NY 10032, USA; 2 Us Helping Us, People Into Living Inc., Washington, DC 20010, USA; dhickson@uhupil.org; 3 Department of Epidemiology, Columbia University Mailman School of Public Health, New York, NY 10032, USA; lt2788@cumc.columbia.edu (L.T.); dd3018@cumc.columbia.edu (D.T.D.)

**Keywords:** transgender women who have sex with men, cisgender men who have sex with men, HIV, Black TWSM, Black cisgender MSM

## Abstract

Most HIV research combines transgender women who have sex with men (TWSM) with cisgender men who have sex with men (MSM), despite emerging evidence of important differences. Using data from The MARI Study, we compared Black TWSM and Black cisgender MSM on personal and ecological factors. Black TWSM reported more unemployment (71.4% versus 51.4%, *p* = 0.015), incarceration (52.4% versus 36.0%, *p* = 0.046), stressful life experiences (median score 135.5 versus 90, *p* = 0.033), and HIV positivity (66.7% versus 22.9%, *p* = 0.008). Further research into the causes and consequences of these differences, and regarding TWSM specifically, is needed.

## 1. Introduction

There is a high burden of HIV among both cisgender men who have sex with men (MSM) and transgender women who have sex with men (TWSM), and racial disparities indicate that Black cisgender MSM and Black TWSM experience the greatest burden of disease. If current trends continue, for example, it is expected that 1 in 2 Black cisgender MSM will acquire HIV in their lifetime, compared to 1 in 11 of their white counterparts [1]. One meta-analysis found 56.3% HIV prevalence among Black TWSM, twice the rate of their white and Hispanic counterparts [2]. In addition to this racial disparity, a geographic disparity has been observed whereby the rate of new HIV acquisitions is 24.5 per 100,000 persons in the Deep South compared to 18.0 per 100,000 in the U.S. overall [3].

In addition to well established psychological and medical differences between TWSM and cisgender MSM [4], emerging research has begun to show differences in socio-demographic characteristics [5], sexual behaviors [6], as well as network characteristics such as sexual network stability [6], factors that may be important to HIV acquisition and transmission. Nevertheless, HIV epidemiologic research has historically combined TWSM with cisgender MSM when study data are analyzed and reported. This has likely occurred because of a lack of appreciation of the potential differences between these groups and the small relative size of the TWSM population in some samples, and has had the effect of conflating any distinctions between these populations. The goal of this analysis was to disaggregate these populations and examine potential socio-demographic, sexual behavioral, stress, socio-cultural, and neighborhood/geographic differences in an existing sample of Black cisgender MSM and Black TWSM in the Deep South.

## 2. Materials and Methods

### 2.1. Study Population

The Ecological Study of Sexual Behaviors and HIV/STI Among African-American MSM in the Southeastern U.S. (known locally as The MARI Study) investigated HIV risks and sexual behaviors among Black cisgender MSM and Black TWSM in Jackson, MS, and Atlanta, GA. Eligibility criteria for the study included self-reporting of African-American or Black race, male sex at birth, 18 years of age or older, residence in the Jackson, MS, or Atlanta, GA metropolitan statistical areas (MSAs), and oral or anal sex with a man in the six months prior to study enrollment. Data were collected between 2013 and 2015, and study recruitment details and procedures have been described previously [7]. Data was collected using audio computer-assisted survey interviewing (ACASI). The MARI Study research protocols were approved by the Sterling Institutional Review Board and all participants provided written informed consent. The secondary analyses reported here were determined to be exempt by the Columbia University Mailman School of Public Health Institutional Review Board.

### 2.2. Socio-Demographic Variables

Participants were asked to report their gender identification; participants reporting female or transgender identity were included in the TWSM group. Socio-demographic variables examined included age (years), ethnicity (not Hispanic or Hispanic/Latino), sexual orientation (gay or homosexual; bisexual; or straight, heterosexual, questioning, or other), highest level of education (high school, GED, or less; some college, trade school, or vocational education; college or higher), current employment status (working full or part time, or unemployed), ever incarcerated (yes or no), and study site (Jackson, MS or Atlanta, GA). Current HIV status was also obtained through laboratory testing (negative or positive).

### 2.3. Sexual Behaviors

Self-reported sexual behaviors assessed in this study were selected to maintain consistency with previously reported analyses [8]. These included alcohol or drug use before or during sex (what constituted sex was left to participants to define, unless otherwise specified) in the previous 12 months (asked separately, yes or no); any condomless anal sex with a casual sexual partner in the previous 12 months (yes or no; inconsistent condom use defined as: most of the time, about half the time, rarely or occasionally, or never); six or more casual male sexual partners in the previous 12 months (yes or no); not having asked the last casual sexual partner’s HIV status (yes or no); and having participated in a sex party or orgy (yes or no).

### 2.4. Stress and Socio-Cultural Characteristics

Life stress was measured through an adaptation of the Holmes-Rahe Life Stress Scale, an 11-item questionnaire that ascertains stressful life events or changes in the previous 12 months [9]. The modified life stress scale demonstrated good psychometric properties (Cronbach’s α = 0.80). Religious participation was measured with a combination of two items from the Daily Spiritual Experience Scale (DSES) [10]: organized religious activity attendance (not at all, less than once a year, a few times a year, a few times a month, at least once a week, nearly every day; scored from one to six), and private religious activity (never, less than once a month, once a month, a few times a month, once a week, a few times a week, once a day, more than once a day; scored from one to eight); this combined measure was then dichotomized at the median into low and high religious participation. The religious participation indicator demonstrated good psychometric properties (Cronbach’s α = 0.69).

### 2.5. Neighborhood Characteristics

The neighborhood problems scale measures the occurrence of six neighborhood issues using a 4-point Likert scale: excessive noise, heavy traffic or speeding cars, lack of access to adequate food and/or shopping, lack of parks and playgrounds, trash and litter, and lack of sidewalks or poorly maintained sidewalks [11]. The neighborhood social cohesion scale uses 5-point Likert items to measure perceptions regarding neighborhood closeness and the degree to which people in the neighborhood trust each other, share the same values, and are willing to help each other [11].

### 2.6. Statistical Analyses

Groups were compared by Fisher’s exact test for discrete variables and Wilcoxon rank sum test for continuous variables. All analyses were conducted using SAS software Version 9.4 (SAS Institute Inc., Cary, NC, USA).

## 3. Results

The analysis sample included 42 Black TWSM and 543 Black cisgender MSM. Black TWSM were more likely to be enrolled at the Atlanta site compared to Black cisgender MSM (71.4% versus 40.0%, *p* < 0.001, see Table 1). Compared to Black MSM, Black TWSM differed significantly on most of the socio-demographic characteristics examined: they were older (median age 31.5 versus 26 years, *p* = 0.006), more likely to identify as heterosexual (35.7% versus 5.7%, *p* < 0.001), more likely to report being unemployed (71.4% versus 51.4%, *p* = 0.015), and more likely to report having ever been incarcerated (52.4% versus 36.0%, *p* = 0.046). HIV prevalence was also significantly higher in the Black TWSM as compared to their Black cisgender MSM counterparts (66.7% versus 22.9%, *p* = 0.008).

Rates of sexual behaviors associated with HIV risk were high in the overall study population (e.g., 49.3% reported using alcohol and 37.4% reported using drugs before or during sex in the previous 12 months, and 39.9% reported having condomless anal sex with a casual partner in the previous 12 months), but did not significantly differ between groups (*p* > 0.05). However, Black TWSM were slightly more likely to report having six or more casual sex partners within the previous 12 months (28.6% versus 17.5%, *p* = 0.09), though this difference was not statistically significant.

Black TWSM reported more stressful life experiences in the previous 12 months as compared to Black cisgender MSM (life stress scale median score of 135.5 versus 90, *p* = 0.033). Rates of religious participation were lower in Black TWSM (43.9% categorized as high religious participation versus 55.9%), although this difference was not statistically significant. Measures of neighborhood problems and neighborhood social cohesion did not differ between Black TWSM and Black cisgender MSM in the study sample.

## 4. Discussion

In The MARI sample, Black TWSM were observed to have higher rates of indicators of low socioeconomic status, marginalization, and stress: they were more likely than Black cisgender MSM to be unemployed, have significantly elevated rates of previous incarceration, and have experienced more stressful life events. All of these factors could contribute to increased HIV risk, and in fact the HIV prevalence among Black TWSM was markedly higher than that among Black cisgender MSM. Surprisingly, we did not observe any statistically significant differences in the reported sexual behaviors between the groups, and they also did not differ in terms of their level of religious participation or the social and physical characteristics of their neighborhood. The observation that structural factors may be responsible for elevated HIV risk, rather than individual behaviors, is consistent with findings in other studies that highlight the importance of non-traditional risk factors such as access to healthcare and disclosure of sexual minority status [12,13].

Though The MARI Study data offers a valuable look at Black TWSM and cisgender MSM in the Deep South, this analysis has several important limitations. First, the data was not collected for the purpose of comparing subpopulations based on gender identity. Since the number of TWSM in the study was small, the power to detect differences between this group and the cisgender MSM in the study may be limited. Despite this limitation, we did observe critical differences between the groups, and additional studies are likely to uncover additional characteristics that represent distinct features of the Black TWSM population. Second, though numerous recruitment strategies were used to enroll participants into the study (i.e., recruitment through community sampling, a geosocial-networking app, and word-of-mouth referrals from participants and community-based organizations), these methods might lead to homogeneity among participants on neighborhood characteristics. Drawing the sample from connected neighborhoods and networks might explain the similarities seen in neighborhood problems and neighborhood social cohesion between the groups. Finally, though The MARI Study data offers information about often understudied groups, especially in the Deep South, other sexual and gender minority populations outside of Black TWSM and cisgender MSM were not represented. We do not know if these results generalize across other diverse populations in terms of sexual behavior and gender identity. However, the high burden of HIV among Black TWSM in particular makes it essential that research explores key features of this group.

With The MARI Study data we were able to examine characteristics beyond those included in other studies and identified factors that make Black TWSM distinct from Black cisgender MSM, despite the small proportion of Black TWSM in our sample. Future research should be designed with the power to detect differences between these groups on other measures that might have an impact on HIV risk. In addition, studies designed to look specifically at TWSM are vitally important, and a few are underway [14,15]. Given that The MARI Study data points to differences in non-traditional factors that have also been observed in previous studies, this work must look beyond individual behavioral differences and consider structural and ecological sources of risk.

## 5. Conclusions

HIV epidemiologic research studies have historically either combined TWSM with cisgender MSM or ignored TWSM entirely, making the implicit assumption that risk factors for HIV acquisition and/or transmission do not differ between these groups. However, prospective studies that have begun to disaggregate these populations suggest that this practice might obscure differences that are important in explaining the high HIV burden in both cisgender MSM and TWSM [5,6]; this study contributes to this emerging understanding.

## Figures and Tables

**Table 1:** Socio-demographic, sexual behavioral, stress, socio-cultural, and neighborhood/geographic characteristics among The MARI Study participants, by gender identity ^a^.

Variable	Statistic/Levels	Black Cisgender MSM (n = 543)	Black TWSM (n = 42)	*p*-Value ^b^
**Socio-Demographic Characteristics**				
Age (years)	Median (Q1, Q3)	26 (21, 38)	31.5 (25, 45)	0.006
Ethnicity	Not Hispanic	528 (97.6%)	41 (100.0%)	0.61
	Hispanic/Latino	13 (2.4%)	0 (0.0%)	
Sexual orientation	Gay/homosexual	369 (68.0%)	24 (57.1%)	<0.001
	Bisexual	143 (26.3%)	3 (7.1%)	
	Straight/heterosexual, questioning, other	31 (5.7%)	15 (35.7%)	
Highest level of education	High School/GED, or less	219 (40.4%)	18 (42.9%)	0.68
	Some college, trade school, vocational	214 (39.5%)	14 (33.3%)	
	College, or higher	109 (20.1%)	10 (23.8%)	
Employment	Working full/part time	263 (48.6%)	12 (28.6%)	0.015
	Unemployed	278 (51.4%)	30 (71.4%)	
Ever incarcerated	No	345 (64.0%)	20 (47.6%)	0.046
	Yes	194 (36.0%)	22 (52.4%)	
Study site	Jackson, MS	326 (60.0%)	12 (28.6%)	<0.001
	Atlanta, GA	217 (40.0%)	30 (71.4%)	
HIV status	Negative	219 (77.1%)	3 (33.3%)	0.008
	Positive	65 (22.9%)	6 (66.7%)	
**Sexual Behaviors**				
Alcohol before/during sex (12 months)	No	266 (49.8%)	26 (61.9%)	0.15
	Yes	268 (50.2%)	16 (38.1%)	
(Drugs before/during sex (12 months)	No	331 (62.1%)	29 (69.1%)	0.41
	Yes	202 (37.9%)	13 (31.0%)	
No condom anal sex casual partner (12 months)	No	314 (59.9%)	26 (61.9%)	0.87
	Yes	210 (40.1%)	16 (38.1%)	
Number casual sex partners 6 + (12 months)	No	448 (82.5%)	30 (71.4%)	0.09
	Yes	95 (17.5%)	12 (28.6%)	
Did not ask last casual sex partner HIV status	No	281 (66.0%)	16 (55.2%)	0.23
	Yes	145 (34.0%)	13 (44.8%)	
Participated in sex party/orgy (12 months)	No	450 (84.4%)	39 (92.9%)	0.18
	Yes	83 (15.6%)	3 (7.1%)	
**Stress and Socio-Cultural Characteristics**				
Life stress scale	Median (Q1, Q3)	90 (0, 164)	135.5 (48, 251)	0.033
Religious participation	Low	228 (44.1%)	23 (56.1%)	0.15
	High	289 (55.9%)	18 (43.9%)	
**Neighborhood Characteristics**				
Neighborhood problem scale	Median (Q1, Q3)	11 (7, 15)	12 (9, 16)	0.27
Neighborhood social cohesion scale	Median (Q1, Q3)	13 (12, 14)	13 (12, 14)	0.93

^a^ Gender identity was defined as cisgender men who have sex with men (MSM) or transgender women who have sex with men (TWSM). ^b^ A Fishers exact test was used for discrete variables, and a Wilcoxon rank sum test was used for continuous variables.

## Data Availability

No new data were created or analyzed in this study. Data sharing is not applicable to this article.
